# Covering Materials Incorporating Radiation-Preventing Techniques to Meet Greenhouse Cooling Challenges in Arid Regions: A Review

**DOI:** 10.1100/2012/906360

**Published:** 2012-05-02

**Authors:** Ahmed M. Abdel-Ghany, Ibrahim M. Al-Helal, Saeed M. Alzahrani, Abdullah A. Alsadon, Ilias M. Ali, Rabeh M. Elleithy

**Affiliations:** ^1^Agriculture Engineering Department, College of Food and Agriculture Sciences, King Saud University, P.O. Box 2460, Riyadh 11451, Saudi Arabia; ^2^Chemical Engineering Department, College of Engineering, King Saud University, Riyadh 11451, Saudi Arabia; ^3^Plant Production Department, College of Food and Agriculture Sciences, King Saud University, Riyadh 11451, Saudi Arabia

## Abstract

Cooling greenhouses is essential to provide a suitable environment for plant growth in arid regions characterized by brackish water resources. However, using conventional cooling methods are facing many challenges. Filtering out near infra-red radiation (NIR) at the greenhouse cover can significantly reduce the heating load and can solve the overheating problem of the greenhouse air. This paper is to review (i) the problems of using conventional cooling methods and (ii) the advantages of greenhouse covers that incorporate NIR reflectors. This survey focuses on how the cover type affects the transmittance of photosynthetically active radiation (PAR), the reflectance or absorptance of NIR and the greenhouse air temperature. NIR-reflecting plastic films seem to be the most suitable, low cost and simple cover for greenhouses under arid conditions. Therefore, this review discusses how various additives should be incorporated in plastic film to increase its mechanical properties, durability and ability to stand up to extremely harsh weather. Presently, NIR-reflecting covers are able to reduce greenhouse air temperature by no more than 5°C. This reduction is not enough in regions where the ambient temperature may exceed 45°C in summer. There is a need to develop improved NIR-reflecting plastic film covers.

## 1. Introduction

The main technical problem of greenhouses is to maintain air temperatures and relative humidity that are favorable for plant growth in the greenhouse. This can be achieved by heating greenhouse air in winter and cooling it in summer. In cool regions, the technology for heating greenhouses is well established and straightforward. However, in hot and sunny regions, cooling the greenhouse air is a more difficult challenge than heating due to the fact that advances in the greenhouse cooling technology are still limited compared with heating systems. In addition, cooling systems are more expensive to install and operate than heating systems. Several efforts have been made worldwide to adopt greenhouses to hot and sunny climate conditions. Even though, an extensive survey was provided for the greenhouse cooling technologies worldwide [1, 2]; however, their survey focused on greenhouses located in tropical and subtropical regions and those located in regions characterized by mild climate such as the south part of Europe. However, in regions characterized by an arid climate with brackish water resources, a discussion for adapting an adequate cooling technique that can be used for greenhouses is still missing. Climate in arid regions is characterized by hot and long summer seasons (the ambient temperature exceeding 45°C at around noon in summer), high solar radiation flux (the daily solar radiation integral reaches to 30 MJ m^−2^), dusty and dry weather (relative humidity of the ambient air drop below 10% at around noon), and water resources being scarce and brackish (salty). Under such conditions use of conventional cooling methods for greenhouses worldwide faces many difficulties that will be discussed later. In addition, such harsh weather conditions negatively affect the plastic films used for covering greenhouses and rapidly degrade their optical and mechanical properties. Apart from the composite cooling systems such as earth-to-air heat exchanger, methods commercially used to reduce the inside greenhouse air temperature under hot and sunny climatic conditions can be divided into three main categories: ventilation, evaporation, and heat prevention. A survey of the literature and of greenhouse growers revealed that neither ventilation nor evaporation is sufficient for cooling greenhouses in arid regions. However, preventing heat from entering the greenhouse is the most appropriate technique for cooling greenhouses. The objectives of this survey were to (i) summarize the conventional methods used for cooling greenhouses worldwide and the limitations to applying these methods to greenhouses in arid regions, (ii) discuss heat prevention methods, especially those that use covering systems able to block near infrared radiation (NIR: 700–2500 nm) as one of the most suitable techniques for greenhouse in arid regions, (iii) review the NIR-reflecting covers that are available in the literature in terms of their transmittance of photosynthetically active radiation (PAR: 400–700 nm) and their reflectance of NIR, and (iv) survey NIR-reflecting film additives that increase the film's mechanical properties, durability, and resistance to harsh and dusty weather.

## 2. Greenhouse Cooling Challenges in Arid Regions

### 2.1. Ventilation

 Ventilation is accomplished by replacing the existing hot air in the greenhouse with cooler air from the outside. If the outside temperature is low enough, and if the temperature inside the greenhouse is not too high, warm air is exhausted passively through the greenhouse vents (natural ventilation). The effectiveness of this system depends on the temperature difference between inside and outside the greenhouse (bouncy effect) and on the wind speed outside the structure (wind effect). At low wind speed, exhaust fans are needed to induce air circulation through the vents (forced ventilation). Many researchers have studied the phenomenon of natural and forced ventilation in agricultural greenhouses [3–8]. In areas where summer is not severe and the maximum ambient temperature remains less than 33°C, ventilation systems can work well [1]. In extreme environments, such as in the Arabian Peninsula, the ambient temperature in summer generally exceeds 45°C. Thus, it is impossible to apply a ventilation method (natural or forced ventilation) because it replaces the overheated greenhouse air with a very hot ambient air. In addition, the relative humidity of air in such regions is always low (less than 15%) especially in the summer season. Thus, ventilation methods cannot provide adequate cooling capacity and suitable environment for plant growth in greenhouses.

### 2.2. Evaporative Cooling

Evaporative cooling involves no change in the enthalpy of air/water vapor mixture. It is known that if one gram of water evaporated into 1 m^3^ of air space, under atmospheric pressure, this can reduce air temperature by about 2.5°C. This technique can lower the greenhouse air temperature significantly below the ambient air temperature and can enhance the relative humidity in the greenhouse to the required and desired levels by controlling the evaporated cooling water. Therefore, under arid climatic conditions, evaporative cooling is the most efficient cooling method for controlling the temperature and humidity in greenhouses. Commercial evaporative cooling systems commonly used for cooling greenhouses are the well-known wet-pad and fan system [9–11] and fogging system [12–17]. In one of the earlier studies, Landsberg et al. [18] reported that for a freely transpiring crop in an evaporatively cooled greenhouse, the air temperature can be reduced by 8–12°C, on average, under high ambient temperature and radiation intensities. A recent experimental study [19] found that, under extreme arid summer conditions in the central region of Saudi Arabia, (the outside radiation flux was about 1100 W m^−2^ at noon), a single stage evaporative cooler having a new pad could reduce the greenhouse air temperature by about 12°C and increased the relative humidity by about 30%. These systems operate efficiently in an arid climate if a pure and fresh water resource is available for wetting the pad or for pumping through the nozzles of a fogging system. However, the Arabian Peninsula is characterized by a lack of water resources and the salinity of the water is very high. This causes a fast deterioration in the cooling performance of the wet-pad fan systems due to clogging the pad as affected by salt buildup on the pad surfaces that also restricts the air flow. Clogged pads were found to reduce the cooling system performance significantly, increase the electric energy consumed by the fan motors by about 22%, increase the inside greenhouse air temperature up to 55°C, and reduce the relative humidity below 10% [11]. Brackish water cannot be used for operating a fogging system; it blocks the fogging nozzle orifices and a costly water treatment is necessary.

For these reasons, neither ventilation nor evaporative cooling techniques are suitable in a region characterized by an arid climate, extensive solar radiation, and high salinity of water resources. Accordingly, deflecting the heat load at the greenhouse cover may provide a promising solution for the overheating problem of the greenhouse air in hot summer seasons under such conditions. This technique will be discussed in the following section.

### 2.3. Heat Prevention

 The spectral distribution of global solar radiation flux, on a horizontal surface, that is incident on or transmitted into a greenhouse can be divided into ultraviolet radiation (UV: 200–400 nm; about 5% of global solar radiation), visible light or photosynthetically active radiation (PAR: 400–700 nm; about 45%), and near infrared radiation (NIR: 700–2500 nm; about 50%). Over the entire spectrum range of global solar radiation, only the PAR is very important for plant growth; it is absorbed by plants for photosynthesis. Therefore the desirable covers for greenhouses in hot and sunny regions should highly transmit PAR and reject NIR and UV. The contribution of UV radiation to greenhouse heating load is insignificant because it represents only 5% of the global radiation. However, the UV should be rejected because it may harm crops and increase insect population and fungal and diseases. Recently most of the plastic films used for covering greenhouses are UV-absorbers. This reduces pest and disease impact on the crops and lower pesticide load and costs. The NIR is less absorbed by the plants but it is absorbed mainly by the greenhouse floor soil, installations, and construction elements of the greenhouse. Then it is released again to the greenhouse air as convected heat that increases the greenhouse air temperature. Accordingly, NIR is the main source of heat load that should be removed from the greenhouse air to prevent the overheating problem in summer in many sunny areas worldwide.

Through heat prevention methods, the radiative heat load can be eliminated or reduced before entering the greenhouse by either absorbing and/or reflecting a portion of the incident radiation on the greenhouse cover. This is accomplished by using commercial shading devices (curtains, clothes, or plastic nets) or by using a radiation filtering roof (blocking the NIR via reflection or absorption and transmitting the PAR).

#### 2.3.1. Shading

Shading the roof of a greenhouse is usually performed by various conventional methods such as whitening the roof [20, 21], external shade cloths [22–25], deploying plastic nets of various colors, and movable refractive screens or curtains [10, 26–33]. Whitening (white shading paint) can be achieved by spraying the exterior cover surface with an aqueous solution of hydrated Calcium oxide (Ca(OH)_2_). Whitening the greenhouse roof is inexpensive, has positive effects on both microclimate and crop behavior, and can be considered an efficient means for alleviating the large heat load during summer [20, 21]. However, it reduced the average greenhouse transmittance to solar radiation from 0.62 to 0.31 [20]. The whitening is washed away if rains fall over the greenhouse and its shading density cannot be changed once applied. The external shade cloth is usually applied by deploying wet or dry shade cloths on the outer surface of the greenhouse roof. An external or internal shade can also be obtained by using movable plastic nets, curtains, or refractive screens applied above or below the roof of the greenhouse. All shading methods are to regulate the amount of solar energy entering the greenhouse and reduce the heating load in summer. Besides protecting plants against excessive heat load, shading significantly reduces the water requirement in arid regions [34]. Disadvantage of shading system that used curtain or screen below the roof of the greenhouse is that when the curtain or screen is fully deployed, it will decrease the effectiveness of the natural roof ventilation and negatively affect the greenhouse microclimate. Moreover, presence of shading materials deployed in the greenhouse absorbs a portion of solar radiation, reemits it again in the greenhouse, and reflects back a portion also inside the greenhouse. Therefore, the effect of internal shading on reducing the greenhouse air temperature is expected to be small. All the aforementioned shading methods significantly reduce solar radiation across the whole solar spectrum including the PAR (400–700 nm) which is essential for plant growth. Therefore, recent studies have focused on developing more selective covers that can transmit PAR and block NIR.

#### 2.3.2. Radiation Filters

For greenhouses in hot and sunny regions, scientists and companies have worked for many years to develop greenhouse covering systems able to reduce the heat load as well as the air temperature in the greenhouses. Among the previous studies, two systems have been introduced for filtering out the incident solar radiation at the greenhouse cover, that is, a double-layer, fluid-roof cover that includes a liquid radiation filter and a solid-roof such as glass or plastic films that includes NIR reflectors.

**Fluid-Roof Covers**

One of the first attempts to eliminate the maximum temperature of air in a glasshouse was carried out by Morris et al. [35] by flowing a water film of 0.5 mm thickness on the roof, and a drop of 4-5°C in the inside air temperature could be achieved. A water film up to 10 mm thickness did not reduce the PAR transmission significantly; however, it blocks (via absorption and reflection) only about 5% of the NIR [36]. Therefore, the cooling effect of a water film flowing on the greenhouse roof is unable to provide enough drop in the greenhouse air temperature in regions where summer air temperatures reach 50°C. More selectively, instead of pure water, a solution of CuSO_4_ in water has been used to selectively transmit most of the PAR and absorb most of the NIR (called liquid radiation filter, LRF). A concentration of 1.5%~2% CuSO_4_-water solution as LRF flowing through a hollow-channeled, rigid plastic (polycarbonate or acrylic) roof of semiclosed greenhouses has been examined via simulation studies and practical tests [37–42]. Based on these studies, the fluid-roof covers can remove, via absorption, more than 50% of solar energy incident on the greenhouse cover; thus the radiation heat load in the greenhouse can be reduced. At solar noon, the fluid roof cover could maintain the inside air temperature about 5°C below the outside temperature [41]. The heat absorbed by the LRF can be stored and used for heating the greenhouse air at night or for other public purposes. The ventilators in the fluid-roof greenhouse can be kept closed during most of the daytime, so that CO_2_ can be enriched with little or no loss to the outside. Although the fluid-roof cover prevents a considerable amount of heat from entering the greenhouse, its transmission of PAR is relatively low because of the complex structure. For example, for double layers made of polycarbonate sheets (1.5 mm thick) and filled with liquid radiation filter (1.5% CuSO_4_-water solution), the transmittance of PAR did no exceed 63% [42]. Previous studies of fluid-roof greenhouses have been conducted only on experimental scales because (i) the structure of the roof is complex, that is, hollow-channeled, made from rigid plastic sheet, (ii) it requires considerable construction investment, one meter square of the roof costs about 50 US $, (iii) such greenhouses need a heat exchanger to cool the LRF in summer, and (iv) there are possible hazards due to the use of toxic copper salts as LRF, so that the cover must be free of leaks. Such limitations make fluid-roof greenhouses impractical on a large scale. Thus there is a need for alternative covering materials (i.e., plastic films or rigid sheets, glass, movable screens, and paints) that can selectively transmit the PAR and reflect, instead of absorb, the NIR in order to solve the overheating problem of greenhouse air.

**NIR-Reflecting Film Covers**

To avoid the complexity, possible hazards and the high cost of the fluid-roof covers, many studies have investigated film covers (plastic sheets or glass) that can provide a cooling effect in greenhouses. To accomplish this, plastic films for greenhouse coverings under harsh weather conditions have to match the following requirements: (1) high transmission of PAR, which is the most important portion of the spectrum for plant growth; (2) high reflection of NIR, which is the main source of heat load that needs to be removed from the greenhouse; (3) sufficient light scattering or diffusive effect, which prevents direct radiation which could damage the plants by rising the tissue temperature; (4) resistance to dust accumulation, which is important because it could affect the transmission of light, especially PAR; (5) drip resistance, which prevents the formation of small water droplets on the covering film and the risk of them falling onto the plants and causing fungal disease; (6) high mechanical strength to prevent wind damage; (7) High durability to retard degradation by UV radiation, chemical (pesticide), and high temperature; (8) cooling effect, especially during summer season; and (9) Low cost.

This survey covers only three of the previous requirements: cooling effect, high mechanical properties, and durability against photodegradation (UV radiation). These three properties are the most important for greenhouses in arid areas like the Arabian Peninsula. Monolayer PE films, especially Low-Density Polyethylene (LDPE) films, will be considered here because of their low cost and popularity.

**(i) Cooling Effect**

Japanese companies (e.g., Asahi Glass Green-Tech Co. Ltd and Mitsui Chemicals Inc.) have started since 2000 to develop different types of fluoropolymers and polyethylene-(PE-) based films and acrylic-based rigid sheets with NIR-reflection additives. Samples of these products have been evaluated to compare their suitability for covering greenhouses in hot regions with fluid-roof covers (e.g., a polycarbonate panel filled with 1.5% CuSO_4_-water solution) [43]. The results indicated that the transmittance of the new products to PAR was in the range from 0.62 to 0.72 compared to 0.63 for the fluid-roof cover. And reflectance of these products to NIR was in the range from 0.37 to 0.54 compared to 0.66 for the fluid-roof cover. To examine the effects of these films in the greenhouse environment, a simulation study was conducted to examine the effects of three types of NIR-reflecting plastic film covers and a fluid-roof cover (polycarbonate panel filled with a 1.5% CuSO_4_-water solution) on air, plant and, soil temperatures in Japan [44]. Closed fluid-roof and naturally ventilated plastic film greenhouses with moist soil were evaluated at different plant densities under hot sunny days. The results showed that at low plant density corresponding to a leaf area index (LAI) of one, the naturally ventilated greenhouse covered with the NIR-reflecting plastic films can keep the inside air temperature equal to the outside temperature. However, at high plant densities (LAI = 4-5), these covers reduced the inside air temperature by about 3°C lower than the outside ambient temperature due to the effect of evapotranspiration. The plant temperature in the closed fluid-roof greenhouse was unacceptably high at high plant densities. The authors suggested the use of the developed NIR-reflecting plastic films to cover a naturally ventilated greenhouse rather than the fluid-roof covers, with a complex and expensive structure, in a region where the temperature and the solar radiation flux are not very high. They also emphasized the need for further studies to improve the films' ability to reflect more NIR and to transmit more PAR.

Pearlescent pigments have been used to produce the so-called interference films. These films consist of three single, nonabsorbing layers, in which the central layer serves as a substrate. The substrate is a fine platelet, and is covered with a fine coating of metal oxides such as TiO_2_. This makes the interference film reflects the NIR without significantly affecting the transmission of PAR. Two German companies (Hyplast/Klerk's and Merck KGaA) joined together to develop an interference film called Kool Lite/Astrolux. This film was commercialized for climate control in regions with high solar irradiance. In order to obtain a stronger reflection of NIR combined with a higher PAR transmission, Merck KGaA had developed Lite/Astrolux film by increasing the coated layers to six including two TiO_2_ layers, one on each side of the film. The new film was designated as “Kool Lite Plus” and has been evaluated and compared with Lite/Astrolux film and a standard diffusive PE film [45]. Three identical tunnels were covered by the three films in the South of Tunisia in hot summer days. The results showed that the maximum air temperature in the tunnel covered with Kool Lite Plus film was lower than that in the tunnel covered with the reference PE film by about 1-2°C at noon and by about 4-5°C in the early afternoon. The PAR transmittance of the Kool Lite Plus film was almost the same as that of the standard film, which means that the selective pigment added to the new film did not affect the PAR transmission. On the other hand, the Kool Lite Plus film cover reduced the global solar radiation transmittance by 4.3% below the standard film cover. This means that the NIR reflectance of the Kool Lite Plus film was only 4.3% greater than that of the standard PE film.

The NIR can be rejected by applying absorption, reflection, or interference pigments to the polymer during manufacturing the covering materials. Interference pigments reflect the NIR and also the PAR according to the incident angle of solar beam radiation. Therefore, optimizing the orientation of the interference pigment in the polymer is necessary to get the maximum NIR reflection, and maximum PAR transmission through the day. Hoffmann and Waaijenberg [46] discussed the suitability of different materials, including absorption, reflection, and interference pigments for covering greenhouses in tropical and subtropical climate. They suggested that the NIR-reflecting and interference materials are preferable for tropical climates. For subtropical regions, they suggested that the covering materials should combine NIR reflection with interception of far infrared (FIR) to prevent thermal loss at night. However, it is better not to include the absorbing pigments in the covering materials because the absorption process increases the cover temperature. Part of the absorbed energy is emitted as thermal radiation into the greenhouse and will lead to a rising inside air temperature. In addition, the life time of the covering material will decrease.

Runkle et al. [47] investigated the effects of new multilayer NIR-reflecting materials produced by 3 M (St. Paul, MN, USA) on the greenhouse microclimate. The new materials were produced as a solid screen and as a woven curtain. The two materials were compared with a neutral metalized commercial shade-screen (55% shading). The three types were installed 0.5 m below the roof of a greenhouse located in a subtropical climate. Based on the PAR incident outside the greenhouse, PAR transmittance was 0.21 under the commercial shade-screen, 0.41 under the NIR-reflecting curtain, and 0.43 under the NIR-reflecting screen. And the NIR transmittance was 0.2 under the commercial shade-screen, 0.27 under the NIR-reflecting curtain, and 0.28 under the NIR-reflecting screen. Although the new materials transmitted more PAR than the commercial shade-screen, they also transmitted more NIR than the commercial shade-screen. The total energy transmitted under the new NIR-reflecting materials was higher than that under the commercial shade-screen. Therefore, the new materials did not show any significant effect on the plants or inside air temperatures. However, the ratio of PAR to the total energy under the NIR-reflecting materials was higher than that under the commercial shade-screen.

Hemming et al. [48] examined two new plastic films that covered greenhouse prototypes. The new films were developed by Oerlemans Int. Co. (The Netherlands) and were PE films containing two different concentrations of NIR-reflecting pigments. The results showed that increasing the pigment concentration decreases the PAR transmittance and increases the NIR reflectance. The PAR transmittances of the new films were 0.804 and 0.77 and the NIR reflectances were 0.29 and 0.36, respectively. The latter film, when used as a greenhouse cover, transmitted 25.1% less NIR than a reference PE film cover without NIR-reflecting pigment, but it also transmitted 9.2% less PAR. The effects of these films as greenhouse covers and the greenhouse configuration on reducing the heat load and on the inside greenhouse air temperature were evaluated experimentally under the tropical climate of Indonesia [49]. Because these films had high NIR transmittances (0.71 and 0.64), they did not significantly reduce the greenhouse air temperature [49].

Hemming et al. [50] conducted a simulation study to quantify the effects of three virtual NIR-filtering methods on greenhouse microclimate, energy saving, and production of tomatoes in The Netherlands. The virtual filtering materials, each of which was designed to filter out 100% or 50% of the incident NIR, were (i) a plastic film that covers the greenhouse, (ii) a movable screen installed horizontally inside the greenhouse, and (iii) an external movable screen that does not limit the ventilation capacity of the greenhouse. In each case they estimated the amount of NIR that needed to be blocked to get positive effects. The simulation was carried out by using a greenhouse microclimate software package [50]. The modeling analysis was performed assuming that the PAR transmittance of the plastic film cover is 0.9 for direct beam solar radiation, and 0.83 for diffuse solar radiation; the PAR transmittance of the internal screen is 0.9 for direct beam solar radiation and 0.95 for diffuse solar radiation and that of the external screen is 0.83 and 0.88, and the NIR is filtered out by reflection. The results indicated that, at noon in the summer months, the plastic film and the external screen filters that reflected 100% of the NIR reduced the greenhouse air temperature by 2°C, reduced the cooling requirement by 50%, and increased the production of tomatoes by 8–12% (on average). Using 50% NIR filters reduced the pervious values by half. The internal NIR-reflecting screen had no advantages, but it increased the greenhouse air temperature by up to 5-6°C during clear summer days at noon. This is because filtration of NIR was inside the greenhouse which is meaningless, and because deploying the screen inside the greenhouse reduced the ventilated air flowing through the roof vents. The study indicated that filtering out the NIR during winter increases the heating load required for greenhouses in The Netherlands and significantly reduces crop productivity. The technical and economical potentials of filtering the NIR depend on the climatic conditions (hot or cold; sunny or cloudy weather) and on the filtration method (permanent or movable NIR-reflector). 

Hemming et al. [51] also measured the spectral short-wave radiative properties of three groups of NIR-reflecting materials to be used as greenhouse covers. The first group was several types of NIR-reflecting glass, each had a different NIR-reflective coating, compared to traditional glass. The second group was several polyethylene (PE) films (i.e., a standard PE film, and the same film with NIR-reflecting and absorbing pigments incorporated in different concentrations) and a commercial transparent screen made from high density polyethylene (HDPEs). The third group was two white washes applied to PE films: the traditional one (Ca(OH)_2_-water solution) and a newly developed one. According to the reported results in [51], the PAR transmittance ranges were 0.73–0.92 for the first glass group, 0.71–0.89 for the second PE films group, and 0.6–0.75 for the third whitening group. The NIR reflectance ranges were 0.58–0.76 for the first glass group, 0.32–0.37 for the second PE films group, and 0.37–0.38 for the third whitening group. The NIR-absorbing pigments used in the PE films decreased the PAR transmittance more than the NIR-reflecting pigments. Whitening, in general, reduced the PAR transmittance significantly. The NIR-reflecting PE films were able to reduce the amount of NIR energy transmission by up to 25% and the NIR-reflecting glasses were able to reduce the amount of NIR energy transmission by 50–70%.

Garcia-Alonso et al. [52] examined two new plastic films as greenhouse covers in Southern Spain. The new films were developed by Repsol YPF Inc. and both were monolayer LDPE with mineral NIR absorbers. One of these films did not block the NIR and did not affect the greenhouse microclimate, while the other film partially blocked the NIR. In summer, the new cover lowered the maximum greenhouse air temperature by about 4.5°C compared to the reference LDPE cover. The new cover significantly improved crop production and quality. However, these products are not yet commercially available and their spectral radiative properties have not been reported.

Lopez-Marin et al. [53] examined the effectiveness of whitening a PE film using Ca(OH)_2_-water solution, a PE film with NIR-reflecting pigment, and a reference PE film without additives for covering identical greenhouses in Southern Spain. The results showed that the whitened cover and the NIR-reflecting cover reduced the PAR transmitted into the greenhouse, respectively, by 24% and 15% compared to the standard PE cover. The whitened cover and the NIR-reflecting cover reduced the NIR transmitted, respectively, by 21.5% and 19.2% compared to the standard PE cover. At around noon, the NIR-reflecting cover and the whitened cover reduced the greenhouse air temperature by 3°C compared to the standard PE cover. The NIR-reflecting and whitened covers had similar beneficial effects on crop production and quality.

Impron et al. [54] studied the effects of three types of plastic films for covering identical greenhouses in a tropical climate. One greenhouse was covered with a conventional PE film to be used as a reference (N0) and the other two greenhouses were covered with PE films with two concentrations of NIR-reflecting pigments (N1 and N2). The measured transmittances of direct beam PAR were 0.89, 0.80, and 0.77, respectively, and the measured reflectances of the direct NIR beam were 0.06, 0.21, and 0.26, respectively. The authors concluded that the variation in cover properties of the greenhouses was too small to show effects in the greenhouse microclimate. The use of NIR-reflecting covers obviously makes sense when a higher amount of NIR is reflected without losing too much PAR. They concluded that more research in this area is needed.

NIR-reflecting materials were also added to shading paints, which can be applied as a removable coating to the greenhouse cover. This type of shading is easy to apply and is useful for greenhouses in northern countries with short summer seasons. In 1997, Tanaka [55] studied the effect of applying NIR-reflecting coating to a PE film-covered greenhouse in Japan. The coated PE film reduced the PAR transmittance by 30% similar to a commercial shading screen that was tested and cut out 60% of the NIR. Hence, the required cooling load of the greenhouse was reduced by 8%. Accordingly, the global PAR transmittance of the coated PE film was estimated to be 0.59 and the global NIR reflectance was estimated to be less than 0.6.

Collaboration between Merck Inc. and the University of Hanover (Germany) led to the development of NIR-reflecting pigments (called ReduHeat) that can be added to shading paint to selectively reflect the NIR and transmit the PAR. Von Elsner and Xie [56] covered tunnels with PE film painted with white shading paint containing different concentrations of ReduHeat and found that both PAR transmission and NIR reflection increased with increasing concentration of ReduHeat. Because of these promising results, ReduHeat has become a commercial product distributed worldwide (e.g., Mardenkro B V, the Netherlands).

Mutwiwa et al. [57] studied the effects of coating the roof of a naturally ventilated greenhouse with ReduHeat pigment on the greenhouse microclimate and plant growth under tropical climatic conditions. Their results showed that such coating lowered the inside air temperature by up to 4°C, reduced the PAR transmittance by about 17%, reduced the NIR (700–1500 nm) transmittance by about 28%, reduced the NIR (1500–2300 nm) transmittance by about 12%, and reduced the global radiation transmittance by about 18%. This means that, for a conventional PE film coated with ReduHeat pigment, the PAR transmittance of the coated film is expected to be around 0.72, the NIR (700–1500 nm) reflectance to be around 0.38, and the NIR (1500–2300 nm) reflectance to be around 0.22. In addition, the heat load and plant water requirement decreased, and the reduction in the PAR transmission had no significant effects on plant growth or production [57]. They concluded that a combination of NIR-reflecting cover and natural ventilation is a promising method for regions with high solar radiation flux throughout the year.

 The NIR-reflecting materials became somewhat available in the forms of (i) transparent sheets (glass or plastic films), (ii) movable screens or nets, and (iii) water-soluble powders (coating paints). The benefits of using such materials for covering greenhouses permanently or seasonally depend on the external climate conditions. For example, using NIR-reflecting materials for covering greenhouses permanently in northern countries may have negative effect on crop growth and productivity in winter months. Kempkes et al. [58] used greenhouse climate models to simulate the effects of the NIR-filtering capacity (0%, 50%, and 100% NIR exclusion) on the greenhouse environment. A heated greenhouse under a Dutch climate and a naturally ventilated greenhouse in the Mediterranean basin were simulated in winter and summer. The aim of their study was to determine the best cooling strategy under a mild climate. The expected benefits (productivity, water use efficiency, and lengthening of the growing season) were quantified in each case. Based on the simulated results, they found that (i) a permanent NIR filter is unsuitable for both heated greenhouses in northern regions and for naturally ventilated greenhouse in areas with mild winters, (ii) an NIR-reflecting cover reduces the plant water requirement, the ventilation rate and CO_2_ concentration in the greenhouse, and (iii) applying NIR-reflecting cover in a mild climate should be coupled with a CO_2_ enrichment facility.

Recently, two NIR-reflecting materials with promising radiative properties have been developed: one is a metallic multilayered material (SOL-MOX Hilite from Bekaert, Zwevegem, Belgium) and the other is a dielectric multilayered based on plastic film (Ebiral from 3 M, St. Paul, USA). These films are very durable and their life time is about ten years [59]. The average PAR transmittances of the dielectric multilayered film and of the metallic multilayered film are 0.85 and 0.75, respectively [59–61]. The NIR reflectance of the dielectric multilayered film was about 0.99 in the wavelength range 900–1200 nm and about 0.15 in the range 1200–2500 nm, while the NIR reflectance of the metallic multilayered film was about 0.8–0.9 in the wavelength range 900–2500 nm [59–61]. These films reflected up to 50% of the incident solar energy (i.e., almost all the NIR). Accordingly, the heat load in the greenhouse was reduced by half [61]. The reflected NIR energy can be concentrated and used to drive photovoltaic (PV) cells to directly generate electric energy. This can be accomplished if the greenhouse cover (NIR-reflecting film) is designed to have several parabolic or circular shapes [60]. The parabolic or circular shapes focus the NIR into lines or small areas. In the focal points, PV cells can be installed to directly convert the NIR-reflected radiation into electric energy. Sonneveld et al. [60] combined this new design with an evaporative cooling system (wet pad and fans). Some of the generated electric energy was used to operate the units of the cooling system and the excess electric energy might be used for a desalination unit or for other public purposes. The optical performance for different types of PV cells was investigated [59] to select the appropriate type for this application. The new design is useful to be successfully applied for a greenhouse with high-grade energy delivery [61]. The energy supply for heating in winter and for cooling in summer can be obtained from this system by efficiently storing the excess electric energy in summer for heating in winter. The system can provide cheaper cooling and energy saving of about 35% compared to heating by fuel [61].

**(ii) High Mechanical Properties**

One of the biggest enemies of greenhouse covering films is the wind. Wind loads increase the tensile and shear stresses on covering films and also mechanically degrade them by abrasion and friction. In particular cases, strong winds mixed with sand (sandstorms) can be especially destructive [62]. The way the film is installed (e.g., with wire supports or clipping) can also cause mechanical stress leading to tearing or rupturing of film [63]. Generally, polyethylene film has a minimum life of 24 months but, recently, polyethylene films with four years life have become available. These films are usually copolymer of polyethylene (PE) and ethyl vinyl acetate (EVA) with incorporation of 1 to 5% vinyl acetate. This formulation has dramatically enhanced the mechanical/physical properties of polyethylene film, including its durability to rupturing and its tearing strength, especially when folded [64].

Research study about LDPE/EVA films has been done by many researchers. Fasce et al. [65] studied the essential work of fracture of photo-oxidized LDPE/EVA films. Aumnate et al. [66] investigated the effect of ethylene vinyl acetate (EVA) on the rheological and mechanical properties of LDPE film used for greenhouse covering system. The mechanical results showed that blend film of LDPE/EVA with 10 wt% EVA content gave the most enhanced mechanical properties as compared to other blends. Hassini et al. [67] investigated the sand-wind effect on the durability of LDPE film (0.2 mm thick) as greenhouse cover, under sub-Saharan climatic condition. They developed the sand-wind simulation in laboratory using special apparatus. From UV-visible spectroscopy analysis, they showed that there was surface roughness modification on LDPE film after sand-wind treatment which leads to dramatically decrease of UV-visible light transmission of LDPE film. Practically, the light transmission decreased by 50% for wavelengths higher than 400 nm. This could be due to the presence of sand-wind particles and surface abrasion caused by the sand-wind on the surface of LDPE film treated. The greenhouse film cover efficiency can be significantly reduced by the sand-wind of very low duration. Therefore, the improvement of LDPE film durability in term of high mechanical properties is needed, and one of the options is to incorporate the LDPE film with EVA. The incorporation of EVA to improve the durability with regard to abrasion by sand-wind effect has been done by Adam et al. [68], though the EVA was incorporated as an individual layer on three-layered greenhouse covering film studied. They showed that the resistance of three-layered film (EVA incorporated) gave a better resistance to the abrasion effect due to sand-wind than normal LDPE (monolayer).

**(iii) Durability from Photodegradation (UV Radiation)**

In order to evaluate the performance of polyethylene film used as a greenhouse cover, several qualitative criteria are used to characterize degradation. The easiest and most common way to evaluate the durability of polyethylene film is to measure the changes in mechanical properties. Particularly, the changes in elongation at break were found to be more sensitive to the degradation process. Therefore this value was usually used as an indicator of degradation [69]. Other measures of degradation include the presence of carbonyl groups, changes in crystallinity, decrease of molecular weight, changes in tensile strength, and increase of density [63].

Degradation of physical properties of PE films due to UV radiation (photodegradation) remains the major cause for limited life of PE film [64]. Photodegradation of polyethylene film occurs from a combination of UV radiation (290–400 nm) absorbed by photoabsorbing chromophores and direct contact with atmospheric oxygen which generates free radicals. The free radicals may act as new chromophores, contacting with the atmospheric oxygen, and may lead to further degradation of PE film. This process is called radical chain-oxidation reaction or photooxidation [63, 70]. One way to slow down the photodegradation of PE film is to add an antioxidant and UV stabilizer. There are three main types of UV-stabilizers: UV-absorbers (UVAs), Hindered Amine Light Stabilizers (HALSs) and Ni-quenchers. Each type works to protect the PE film in a different way in the photodegradation process. UVAs protect the PE film by preferentially absorbing damaging UV rays to prevent them from reaching the chromophores in PE film and releasing them in a nondestructive way in the form of heat energy [71]. HALSs use another way to slow down the photodegradation. They do not provide UV absorption but rather work by scavenging radicals, that is, by trapping and decomposing radical intermediates (free radicals) produced during the photodegradation process [71]. HALSs appear to be the newest and most effective type of UV stabilizer. UVAs should not be applied on thin film (below 100 micron), because they tend to migrate and left from the bulk of PE film within a relatively short time. It has to be taken into account that the UVA content needed to completely protect the surface of PE film is so high that is practically impossible to use such degree. Combining UVA and HALS might be a good way to protect PE film from photodegradation. The use of UVA could minimize the number of free radicals formed during the photo-oxidation process by absorbing the UV-rays, while HALS could control the free radicals that have already formed [71].

Several studies have investigated the performance of commercial UV stabilizers includ UVA and HALS. Al-Salem [72] studied the influence of natural and accelerated weathering test on LDPE film with different types of UV stabilizers and different concentration. In the study, the author has three formulations, namely, (a) which contains UVA additives, for example, Tinuvin 494, Tinuvin NOR 371, and Chimassorb 81 (from Ciba Specialty Chemicals Co, Switzerland); (b) which contains commercial grade light transforming (LT) additive (i.e., Irgastab); and (c) which is combination of UVA and LT additives. The results showed that mechanical properties were improved in the presence of UVA additives. Combination of UVA and LT (C formulation) exhibited a better service life and improvement in exposure duration to natural weathering test. There was also improvement in the time to reach 50% retention of strain compared to formula B. There was not significant changes on % transmission measured; however change in color was totally affected by UVA additives.

Similar research study was done by Basfar and Ali [73]. They performed natural weathering test to study the UV stability of LLDPE and LDPE thin film (0.06 mm). The PE films were formulated with several commercial UV stabilizers (HALS, Chimassorb 81, Tinuvin P, Tinuvin 326) and antioxidants (Irganox 1010, Irgafos 168, Alkanox TNPP). They carried out the test by exposing the prepared PE film direct to sunlight for 365 days. They reported that the PE film with single HALS only has improved the UV stability by about two- to 12-fold over the reference PE, whereas film PE with combination of HALS and UVA gained further improved UV stability over the HALS film and reference film. The improvement was about three times higher in terms of 50% tensile strength retention.

Another research study about UV stability enhancement was done by Salem et al. [74]. They compared between unstabilized LDPE films and LDPE films formulated with HALS (Tinuvin 783 manufactured by Ciba-Geigy) at 0.6 wt%. It was found that the use of HALS has effectively maintained the physical properties (tensile) of the LDPE films during exposure to UV radiation. They also concluded that there was no correlation between mechanical properties and carbonyl group index. There are many other research studies about the use of UV stabilizer (UVA and HALS) to improve the UV stability of PE film from photodegradation [75–78].

Bualek et al. [69] studied the aging of LDPE films for agricultural use in Thailand. In their work, the LDPE plastics were exposed naturally to sunlight radiation as well as in an accelerated weathering test. Under accelerated condition, LDPE films undergo degradation process 7 to 9 times faster than those under natural condition (sunlight radiation exposition). They investigated the effectiveness of some commercial UV-stabilizers in hindering photodegradation of LDPE films. Particularly, the physical properties changes such as elongation at break of many polymers were found to be more sensitive to photodegradation than other analytical methods. In their work, reduction of elongation at break to 2/3 of initial values was used as the standard of failure. The results of accelerated weathering test showed that the incorporation of 0.2% of UV-stabilizer (Chimassorb 944) could improve the life time of LDPE films to approximately 4-5 times longer as compared to that without UV-stabilizer. Additionally, the measurement of infrared spectra has been also conducted to analyze the formation of carbonyl groups as the degradation products. Carbonyl groups were observed in the degradation of unstabilized film, whereas during degradation of LDPE film with UV-stabilizer (Chimassorb 944), no such groups were observed. It indicates that the addition of Chimassorb 944 suppresses the photo-oxidation of LDPE films effectively. In addition, for Tinuvin 622 and Tinuvin 326, smaller carbonyl groups were observed. In other words, for the UV-stabilizers investigated, Chimassorb 944 was found to be more effective in inhibiting photo-oxidation of LDPE film than Tinuvin 622 and Tinuvin 326 (Chimassorb 944 > Tinuvin 622 > Tinuvin 326). Life of LDPE films strongly depended on the thickness of the films. It is because that the photodegradation process takes place from the surface into the bulk of the film.

Many UV stabilizers are commercially available. However, according to a survey, little information is available about their incorporation with local PE film and their use for greenhouse in the Arabian Peninsula. This will be one of our goals to identify such covers for greenhouses.

NIR-reflecting coatings are formulated with special pigments. It has been reported that it was theoretically impossible to predict the IR reflectivity of a pigment. The only and best way to find it is to evaluate the available NIR-reflecting pigment for their IR reflectivity. There are several factors affecting the reflectivity of NIR-reflecting pigment, which are individual pigment selection of NIR-reflective pigments, particle size (at least 0.35 to 0.55 microns), blending NIR-reflective pigments, opacity, and contamination [79].

## 3. Conclusions and Recommendations

 The harsh weather (high air temperature and high solar irradiance) in arid regions having brackish water resources makes it difficult to grow plants in open fields during summer. On the other hand, growing plants in greenhouses requires an efficient cooling system to remove a considerable amount of heat. Under such conditions, conventional cooling methods (ventilation or evaporative cooling using wet pad and fans or fogging systems) face many challenges due to the nature of the weather and water resources. NIR-reflecting plastic films seem to be the most suitable and simplest technique for covering greenhouses under these conditions. NIR-reflecting materials are more efficient than NIR-absorbing materials. The solar spectrum in the wavelength range 700–1100 nm includes most of the NIR and should be reflected out of the greenhouse whereas wavelengths in the range 1100–2500 nm do not carry significant amounts of heat. Many NIR-reflecting and NIR-filtering additives (pigments) are commercially available, but few of them have been investigated. Additionally, their use in greenhouses in the Arab Peninsula with high global radiation is still limited. Therefore, further studies are needed to determine which of these additives are best suited for this region.

In mild climates NIR-filtering is not desirable during winter time. NIR-filtering covers should not be used in heated greenhouses in Northern countries since they cause an undesirable temperature drop. NIR-filtering white washes and movable screens can be applied during periods when needed. However, they still reduce PAR too much. NIR-filtering movable screens should be used for shading outside the greenhouse cover to avoid effecting the greenhouse ventilation. Using NIR-reflecting plastic film for covering greenhouses permanently in the Arabian Peninsula is preferable because the winter season is relatively short and the daytime ambient temperature and solar radiation flux are relatively high. The maximum drop in air temperature that could be achieved under an NIR-reflecting greenhouse cover was 5°C; however, this reduction is not enough in regions having an ambient temperature exceeding 45°C in summer. A perfect NIR-filtering plastic film cover that is suitable for an arid climate with high solar radiation flux is not yet available. More research is needed to develop such kinds of NIR-reflecting PE film covers.

**Figure 1:**
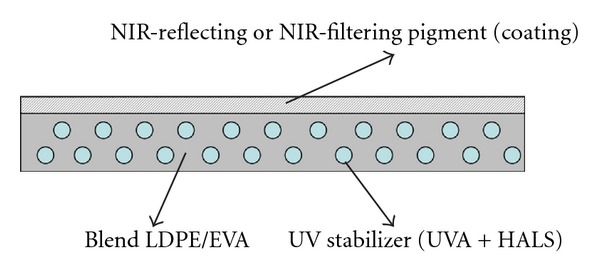
A model PE film for greenhouse covering.

The most important characteristics of greenhouse covering films are ability to provide cooling, high mechanical strength, and durability against photodegradation. A model of such a film covering is shown in Figure 1.

The reflected NIR energy can be utilized by PV cells to generate electric power, which can be used for many purposes including generating distilled water for evaporative cooling systems.
